# Distinct Humoral Response Development to Measles Virus Across Vaccine Platforms

**DOI:** 10.1093/infdis/jiag309

**Published:** 2026-06-17

**Authors:** Andy K P Chan, Jacqueline K Brockhurst, Liting Liu, Shristi Ghimire, Nicole Baumgarth, Diane E Griffin

**Affiliations:** W.Harry Feinstone Department of Molecular Microbiology and Immunology, Johns Hopkins Bloomberg School of Public Health, Baltimore, Maryland, USA; Graduate Program in Immunology, Johns Hopkins University School of Medicine, Baltimore, Maryland, USA; W.Harry Feinstone Department of Molecular Microbiology and Immunology, Johns Hopkins Bloomberg School of Public Health, Baltimore, Maryland, USA; Department of Molecular and Comparative Pathobiology, Johns Hopkins University School of Medicine, Baltimore, Maryland, USA; W.Harry Feinstone Department of Molecular Microbiology and Immunology, Johns Hopkins Bloomberg School of Public Health, Baltimore, Maryland, USA; W.Harry Feinstone Department of Molecular Microbiology and Immunology, Johns Hopkins Bloomberg School of Public Health, Baltimore, Maryland, USA; W.Harry Feinstone Department of Molecular Microbiology and Immunology, Johns Hopkins Bloomberg School of Public Health, Baltimore, Maryland, USA; Graduate Program in Immunology, Johns Hopkins University School of Medicine, Baltimore, Maryland, USA; Department of Molecular and Comparative Pathobiology, Johns Hopkins University School of Medicine, Baltimore, Maryland, USA; W.Harry Feinstone Department of Molecular Microbiology and Immunology, Johns Hopkins Bloomberg School of Public Health, Baltimore, Maryland, USA

**Keywords:** measles virus, humoral immunity, antibody detection, vaccination, challenge study

## Abstract

**Background:**

Different measles vaccine platforms protect against clinical disease, but characteristics of the resulting humoral responses remain incompletely defined.

**Methods:**

In this study, a phage display immunoprecipitation sequencing technology, VirScan, was employed to evaluate measles viral epitope coverage by antibodies following wild-type (WT) measles virus (MeV) infection of rhesus macaques, or vaccination with a live attenuated MeV vaccine (LAMV), the triple measles-mumps-rubella vaccine (MMR), or a novel recombinant MeV hemagglutinin protein–based vaccine (rMeVH). Vaccinated animals were also challenged with WT MeV to measure antibody epitope reactivity during a recall.

**Results:**

The results demonstrate that WT MeV infection elicited a broader epitope reactivity profile than LAMV-containing vaccines. Also, rMeVH vaccination not only resulted in a narrow H protein–specific antibody profile, but epitopes recognized were distinct from LAMV-vaccinated or WT-challenged animals. All vaccine groups expanded the breadth of MeV-specific antibody repertoires following WT MeV challenge. In addition, MeV-specific VarScore, a measure of overall antigen binding, was positively correlated with IgG titer assessed by enzyme-linked immunosorbent assay, as well as antibody avidity and neutralizing titers.

**Conclusions:**

The results demonstrate the distinct effects of vaccination platforms on the breadth and fine specificity of anti-measles–induced humoral responses, and the utility of VirScan for comprehensively assessing antibody epitope targets.

## Teaser

Using VirScan, a phage immunoprecipitation sequencing technology, this study shows that natural measles infection induces broader antibody responses than the live attenuated measles or the novel H recombinant protein vaccines, which target distinct epitope sites.

Since its introduction in the 1960s, the measles virus (MeV) vaccine has reduced measles cases by more than 99% and saved nearly 95 million lives over the past 50 years [1, 2]. Despite the availability of a safe and effective vaccine, measles disease remains a leading cause of vaccine-preventable illness in children, resulting in an estimated 107 500 deaths in 2023 [1, 3]. Disruption to mass vaccination and surveillance campaigns during the COVID-19 pandemic and rising vaccine hesitancy are among the barriers to global measles elimination [1, 4].

MeV is a *Morbillivirus* in the family Paramyxoviridae. Viral particles contain a single-stranded, negative-sense RNA, which encodes 6 structural and 2 nonstructural proteins. The surface glycoproteins hemagglutinin (H) and fusion (F) mediate attachment and entry into host cells, respectively. Other structural proteins include the nucleoprotein (N), matrix (M), phosphoprotein (P), and polymerase (L) [5]. The nonstructural proteins V and C regulate host responses to infection.

In light of increasing measles cases, it is imperative to understand drivers of protective humoral immunity to measles vaccination and infection. MeV-specific antibodies are crucial for protection from subsequent MeV infection. Passive transfer of antibody alone is sufficient to confer protection to MeV-naive individuals [6, 7], and serum neutralizing antibodies, targeting the H and F proteins [8–10], are widely used as the gold standard correlate of protection [11, 12]. Both the live attenuated MeV vaccines (LAMVs) and wild-type (WT) MeV infection result in the production of antibodies directed against N, P, M, and variably L and V, along with H and F [13–17]. WT MeV infection elicits higher titers of MeV-specific immunoglobulin G (IgG) and neutralizing titers than LAMV vaccination [18, 19], but the epitope specificity of antibodies induced by LAMV and other MeV vaccine platforms and their impact on protection is less understood.

The immune response elicited by a novel recombinant prefusion-stabilized MeV H protein vaccine (rMeVH) was recently examined and compared to that induced by the live attenuated measles, mumps, and rubella (MMR) vaccine in rhesus macaques [20]. Both vaccine platforms provided nonsterilizing protection from clinical disease, providing an opportunity to examine the breadth and diversity of epitopes targeted after vaccination or WT MeV challenge.

Previous studies have employed VirScan, a phage immunoprecipitation sequencing technology, to characterize global antibody reactivity to common pathogens following MeV infection [21, 22] and to assess the impact of treatment with the antiviral drug remdesivir on the magnitude and breadth of MeV-specific antibodies in MeV-infected macaques [23]. Here, we used VirScan to describe antigenic epitopes of MeV, mumps virus (MuV), and rubella virus (RuV) in rhesus macaques (*Macaca mulatta*) after MeV vaccination, followed by WT MeV challenge. We compared 4 vaccination strategies: a single dose of intratracheal LAMV (1LAMV), 2 doses of the MMR vaccine (2MMR), 2 doses of the rMeVH vaccine (2rMeVH) [20], or a single dose of the MMR vaccine followed by a single dose of the rMeVH vaccine (1MMR/1rMeVH) (Table 1). Together, these data provide insights into the breadth and diversity of MeV vaccine– and WT challenge–induced humoral immunity.

**Table 1.** Measles Vaccination and Challenge Schedule and Experimental Groups

Group	No. of Animals	Route	Prime (Day 0)	Boost (Day 42)	Challenge (6 Months)
2× MMR	4	Subcutaneous	MMR	MMR	Bilthoven
1**×** MMR/1× rMeVH	4	Subcutaneous/intramuscular	MMR	rMeVH	Bilthoven
2× rMeVH	4	Intramuscular	rMeVH	rMeVH	Bilthoven
1× LAMV	3	Intratracheal	LAMV	–	–
2× PBS	10	Subcutaneous	PBS	PBS	Bilthoven

Abbreviations: LAMV, live attenuated measles virus vaccine; MMR, measles-mumps-rubella vaccine; PBS, phosphate-buffered saline; rMeVH, novel recombinant measles virus hemagglutinin protein–based vaccine.

## MATERIALS AND METHODS

### Study Design

We used both banked samples from previously published studies [20, 24] and novel samples to generate the dataset for this manuscript.

Juvenile (2- to 3-year-old) MeV-seronegative, Indian-origin rhesus macaques were obtained from the Johns Hopkins University (JHU) Primate Breeding Facility. Macaques underwent vaccination and challenge as previously described [20], in addition to a fourth group that received a single intratracheal dose of LAMV (1LAMV) (Table 1). Live attenuated vaccines included the MMR vaccine (MMR II, Merck), containing an Edmonston strain of LAMV (3.0 log_10_ tissue culture infectious dose 50), delivered subcutaneously according to the manufacturer's instructions, or an Edmonston-Zagreb strain of LAMV delivered intratracheally at 10^4^ plaque-forming units (PFU). A dimeric MeV H protein vaccine (Edmonston strain, amino acids 180–617) was delivered intramuscularly at 100 mg MeV H protein mixed with 100 mg of Alhydrogel (InvivoGen) in 100 μL phosphate-buffered saline (PBS) [20] according to the developer's instructions. Approximately 6 months after vaccination, all groups—except for the 1LAMV group—were intratracheally challenged with 10^4^ PFU WT MeV (Bilthoven, genotype C2; gift of Albert Osterhaus, Erasmus University, grown in phytohemagglutinin-stimulated human cord blood cells in 1 mL PBS). At selected timepoints, whole blood was collected from the femoral vein into heparin-coated tubes, and plasma was separated by centrifugation. Plasma was stored at −80°C until analysis.

All studies were performed within an Association for Assessment and Accreditation of Laboratory Animal Care International–accredited facility in accordance with experimental protocols approved by the JHU Animal Care and Use Committee and guidelines in the Animal Welfare Regulations and the Guide for Care and Use of Laboratory Animals.

### VirScan Library Construction and Immunoprecipitation

VirScan was conducted and analyzed as previously published [23]. In brief, macaque plasma samples were diluted 1:20 in PBS. Controls were randomly assigned to a position on a 96-well plate to avoid positional artifacts. The VirScan library was mixed with diluted plasma at 10^5^-fold library coverage. Approximately 20 billion PFU T7 bacteriophages per well were combined with plasma samples and incubated overnight at 4°C. Antibody-bound phages were immune-captured with protein A/G-coated magnetic beads (Dynabeads, Invitrogen) and any unbound phages were washed away. A single-step polymerase chain reaction (PCR) assay added index primers and barcodes for Illumina sequencing. Samples were then pooled and purified using a PCR clean-up kit. Purified PCR products were submitted to the laboratory of Dr. H. Benjamin Larman at the JHU School of Medicine for quantification and sequencing.

### VirScan Data Analysis

Longitudinal plasma samples from each group were subjected to the experimental processes of VirScan with mock control reactions. VirScan output files were generated for each 96-well immunoprecipitation plate, including raw read count and viral aggregate reactivity scores (VarScores) files, from which 3 other datasets were generated: hits/hits fold change (HFC), total peptide hits (TPH), and percentage peptide hits (PPH) files. A detailed explanation is provided in the previous study [23].

In brief, VarScore is a measure of the overall level and breadth of antibody reactivity across the viral species [25] and was generated through the comparison of mean log_2_ fold change values for virus-specific peptides to the distribution of expected mean log_2_ fold change values for randomly selected peptides in VirScan (https://github.com/wmorgen1/ARscore). HFC files computed antibody hits to the peptide, with values >1 suggesting that antibodies significantly bind to and target this viral peptide tile. TPH is defined as the total number of peptide tiles considered as “hits” per viral species or per viral protein in each viral species in each sample; averages of TPH per macaque group are reported as average peptide hits. Last, PPH was calculated by taking the average of TPH by the total number of peptide tiles per viral species. In total, antibody reactivity against MeV, MuV, and RuV was examined at the viral species, viral protein, and the 56-mer peptide tile levels longitudinally for all groups.

### Enzyme Immunoassays

MeV-specific IgG enzyme-linked immunosorbent assay (ELISA), avidity assay, and plaque reduction neutralization test (PRNT) assays were conducted as described previously [20, 24]. WT MeV data were pulled from previously published data [20], while the LAMV data were generated for this manuscript. RuV-specific IgG in plasma was quantified using a human anti-RuV IgG enzyme immunoassay kit according to the manufacturer's instructions (abcam, ab108767*).*

### Statistical and Graphical Analysis

Bioinformatic analysis was conducted using R (version 4.2.2; 2022) and R packages, such as dplyr, ggplot2, ggpubr, tidyverse, readr, stringr, gridExtra, and ggh4x. The Wilcoxon rank-sum test was used to compare values between 2 samples.

## Supplementary Material

jiag309_Supplementary_Data

## Figures and Tables

**Figure 1:**
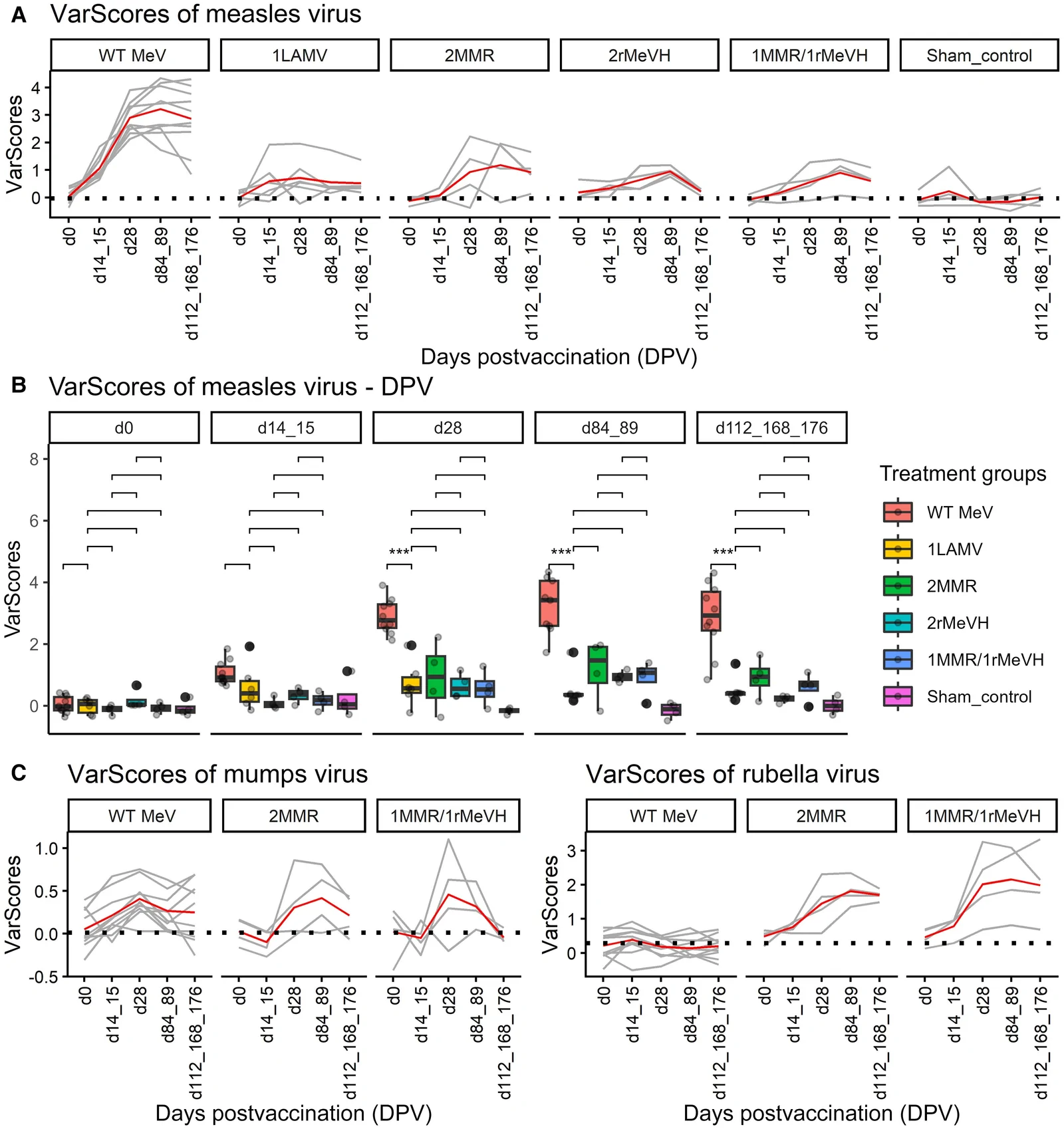
Longitudinal antibody reactivities to MeV, MuV, and RuV in macaques after vaccination. *A*–*C*, VarScores to MeV (*A* and *B*) and MuV and RuV (*C*) in macaques vaccinated with 1 dose of LAMV at day (d) 0 (1LAMV, n = 3), 2 doses of MMR or rMeVH at both d0 and d42 (2MMR or 2rMeVH, respectively, n = 4 per group), or 1 dose of MMR at d0 and 1 dose of rMeVH at d42 (1MMR/1rMeVH, n = 4). Five timepoints per group were captured as indicated. Longitudinal data from WT MeV–infected (n = 10) and sham-infected (n = 4) macaques were also included for comparison. In *A* and *C*, the horizontal dotted lines represent the average longitudinal VarScores from each of the 3 viruses in the sham control group to compute a threshold value, above which a positive infection was identified. The gray lines represent values from each macaque, and the red line indicates the average values from all macaques in each group. In *B*, the boxplot shows the median (center line), IQR (box), and whiskers extending to 1.5× the IQR. Solid and hollow dots represent data points within and outside 1.5× the IQR, respectively. The statistical significance between macaque groups per timepoint was evaluated by Wilcoxon rank-sum test, with nonsignificant results shown by missing asterisks. In *C*, only data from the 2 macaque groups vaccinated against MuV or RuV were included. ***P < .001. Abbreviations: DPV, days postvaccination; IQR, interquartile range; LAMV, live attenuated measles virus vaccine; MeV, measles virus; MMR, measles-mumps-rubella vaccine; MuV, mumps virus; rMeVH, novel recombinant measles virus hemagglutinin protein–based vaccine; RuV, rubella virus; WT, wild-type.

**Figure 2.**
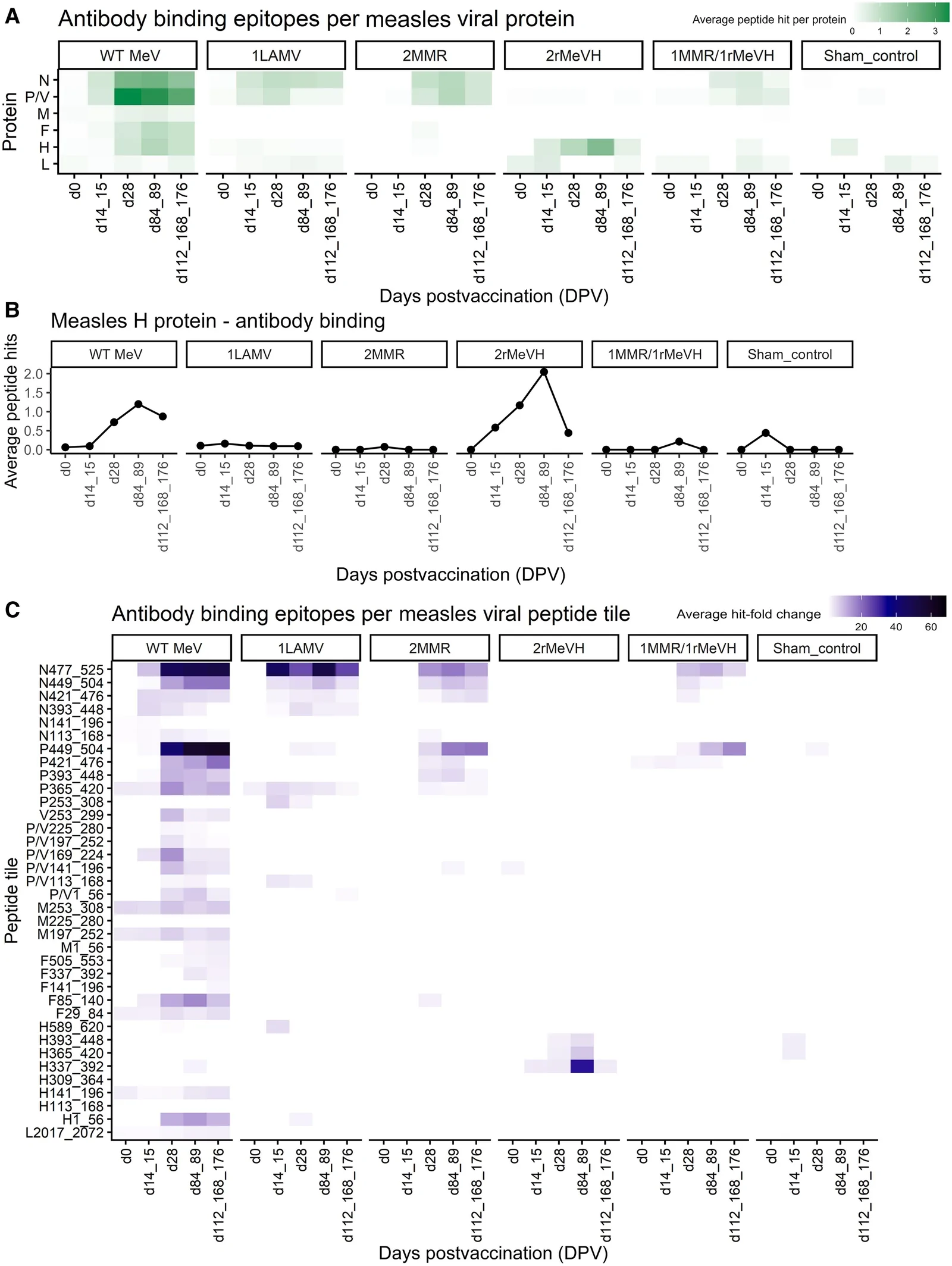
Longitudinal average peptide hits to MeV proteins and peptide tiles after vaccination. Longitudinal average peptide hits to all MeV proteins (*A*), H protein (*B*), and selected viral peptides (*C*) in different vaccinated groups. Since P and V proteins are distinct products from the same gene with P, tiles spanning amino acids 1–231 represent sequences are shared by both P and V proteins (P/V), the V253-299 tile is V-specific, and all remaining tiles beyond amino acid 231 are P-specific. In *B*, average hits to H peptide tiles were generally low (0–0.2), corresponding to recognition of <1 H peptide per animal on average, thus likely reflecting background binding. In *C*, MeV viral peptide tiles were selected if average hit fold change was >20 in any timepoint in any macaque. Five timepoints (day [d] 0, d14/15, d28, d84/89, d112/168/176) were captured per group. Abbreviations: DPV, days postvaccination; LAMV, live attenuated measles virus vaccine; MeV, measles virus; MMR, measles-mumps-rubella vaccine; rMeVH, novel recombinant measles virus hemagglutinin protein–based vaccine; WT, wild-type.

**Figure 3.**
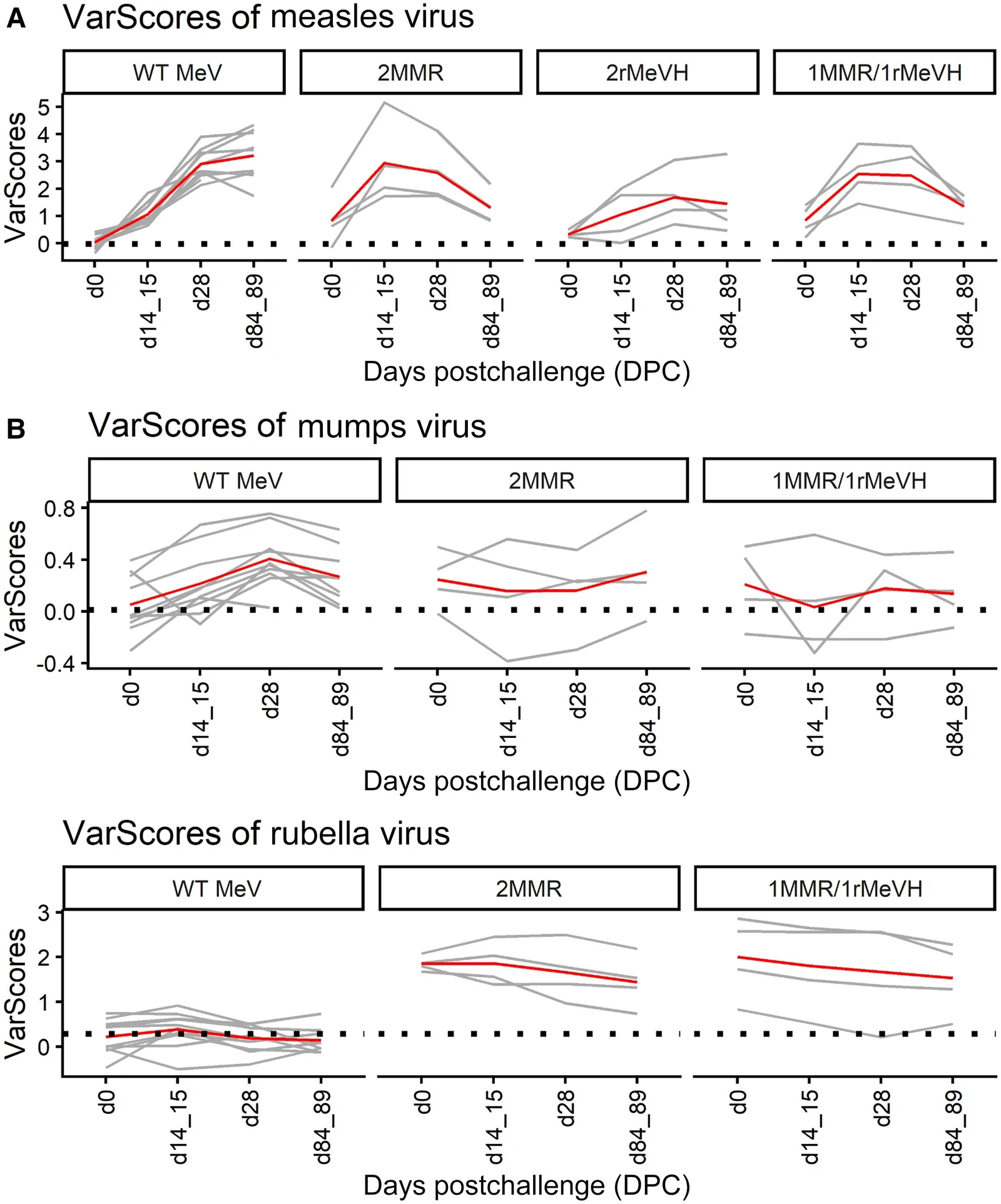
Longitudinal antibody reactivities to MeV, MuV, and RuV in previously vaccinated macaques after WT MeV challenge. VarScores to MeV (*A*) and MuV and RuV (*B*) in previously vaccinated macaque groups: 2MMR, 2rMeVH, and 1MMR/1rMeVH. Longitudinal data from WT MeV–infected macaques were also included for comparison. The horizontal dotted lines in *A* and *B* represent the average longitudinal VarScores from each of the 3 viruses in the sham control group in Figure 1 to compute a threshold value, above which a positive infection was identified. Abbreviations: DPC, days postchallenge; MeV, measles virus; MMR, measles-mumps-rubella vaccine; rMeVH, novel recombinant measles virus hemagglutinin protein–based vaccine; WT, wild-type.

**Figure 4.**
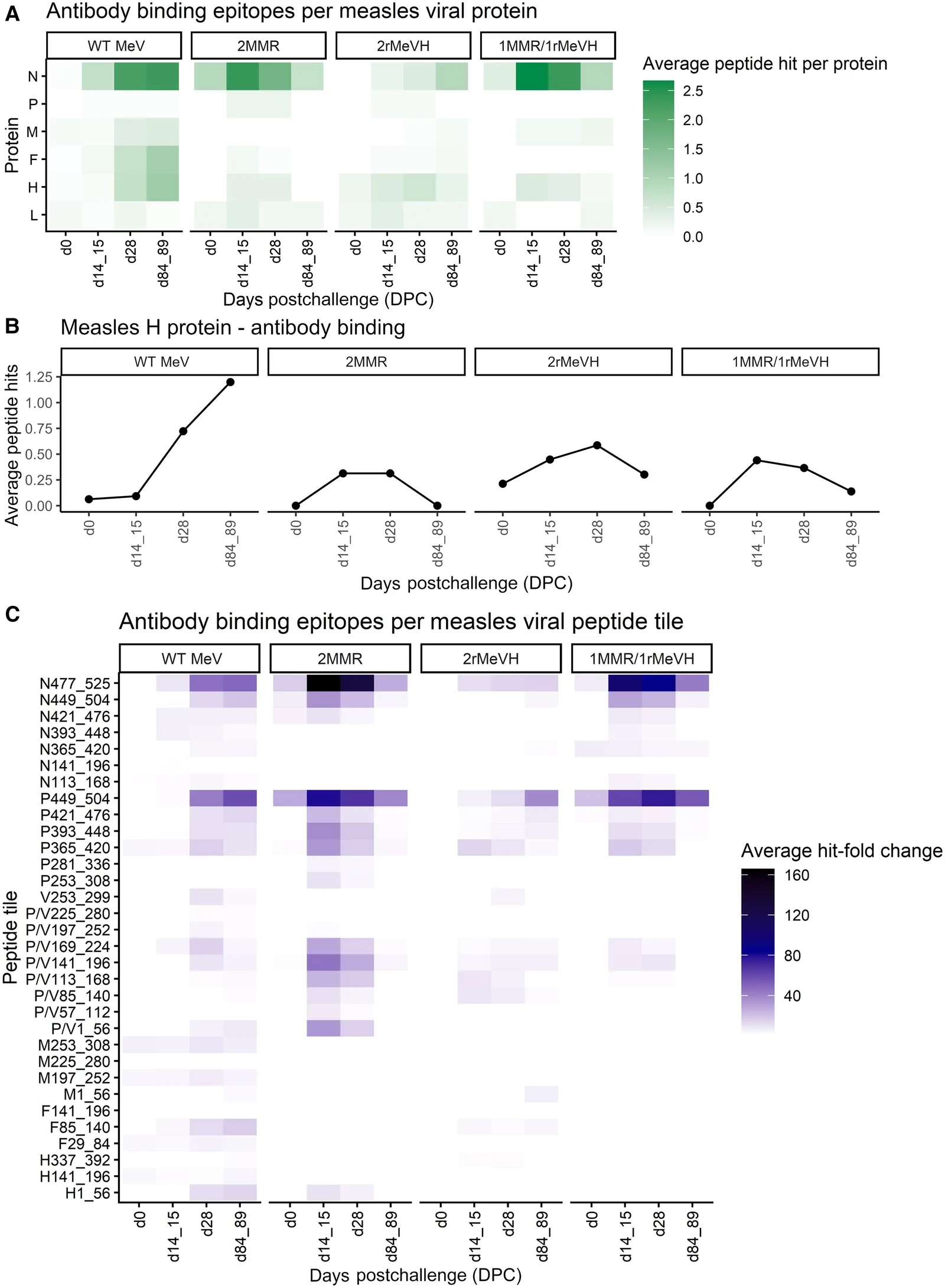
Longitudinal average peptide hits to MeV proteins and peptide tiles in previously vaccinated macaques after WT MeV challenge. Longitudinal average peptide hits to all MeV proteins (*A*), H protein (*B*), and selected viral peptides (*C*), where MeV viral peptide tiles were selected if average hit fold change was >20 in any timepoint in any macaque. Abbreviations: DPC, days postchallenge; MeV, measles virus; MMR, measles-mumps-rubella vaccine; rMeVH, novel recombinant measles virus hemagglutinin protein–based vaccine; WT, wild-type.

**Figure 5.**
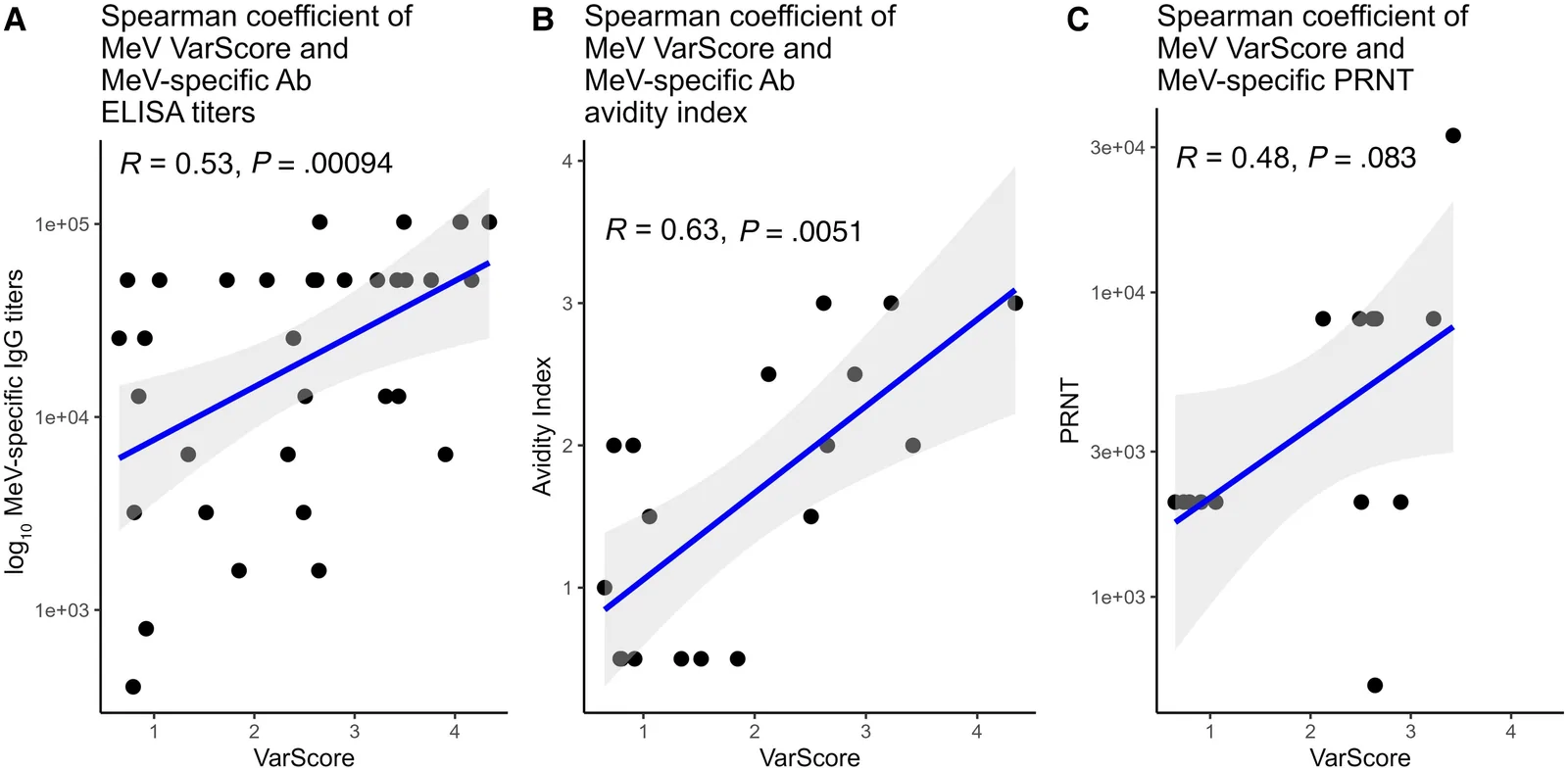
Correlational analysis of MeV VarScore with MeV-specific IgG ELISA titers, avidity indices, and PRNT titers from WT MeV–infected macaques. Spearman correlation was computed between MeV VarScore of WT MeV–infected macaques at 4 longitudinal timepoints (day [d] 14/15, d28, d84/89, and d112/168/176; n = 10) and MeV-specific IgG ELISA titers (*A*), avidity indices (*B*), and PRNT titers (*C*), all of which were derived from previously published data [20, 23]. Abbreviations: Ab, antibody; ELISA, enzyme-linked immunosorbent assay; IgG, immunoglobulin G; MeV, measles virus; PRNT, plaque reduction neutralization test.

## Data Availability

Supporting data values for VarScore and PPH values can be found in the Supplementary Excel file. All R codes and datasets related to data visualization are published in https://github.com/Andy-ChanKP/VirScan_MeV_infection_and_vaccination_analysis (Commit ID: 89e8251).

## RESULTS

### MeV Vaccination Induced Humoral Responses as Measured by VarScores and PPH

First, we measured the cumulative antibody reactivity to all MeV proteins—termed VarScore—in macaques receiving intratracheal LAMV, subcutaneous MMR, or intramuscular rMeVH vaccinations, or intratracheal WT MeV challenge. All vaccinated macaques showed increased MeV VarScores compared to sham-vaccinated controls, with scores peaking between day 28 and days 84–89 postvaccination (Figure 1*A*). MeV VarScores were not significantly different between vaccine groups at any time following primary vaccination at day 0 or boost at day 42, although VarScore peak score was trending higher in 2MMR than other vaccine groups (2MMR at days 84–89, 1.18 ± 1; 1LAMV at day 28, 0.71 ± 0.73; 1MMR/1rMeVH at days 84–89, 0.90 ± 0.58; 2rMeVH at days 84–89, 0.95 ± 0.18) (Figure 1*B*). MeV VarScores for all vaccine groups, at all times postvaccination, were significantly lower those of than macaques that received only WT MeV (peak score at days 84–89, 3.22 ± 0.90), which is consistent with anti-MeV IgG titers reported in the literature [18].

As expected, VarScores for both MuV and RuV increased in animals that received at least 1 dose of MMR, likewise peaking between day 28 and days 84–89 postvaccination. However, MuV VarScore also increased in the WT MeV group (peak score at day 28, 0.41 ± 0.22), likely due to cross-reactivity between MeV antibodies and MuV viral epitopes (Figure 1*C*). The VarScore for RuV was similar between animals in the 2MMR group compared the 1MMR1rMeVH group (Figure 1*C*). VarScores also correlated with PPH, the average percentage of antibody-targeted peptides to the total number of peptide tiles per viral species (Supplementary Figure 1).

### Breadth and Quantity of Antibodies Produced Following MeV Vaccination Differs From WT MeV Infection

We next mapped antibody hits to specific MeV proteins (Figure 2*A* and 2*B*) and peptides (Figure 2*C*) following measles vaccinations or infection. All groups receiving an LAMV-containing vaccine (1LAMV, 2MMR, 1MMR/1rMeVH) developed antibodies targeting the MeV P/V and N proteins, with higher average peptide hits in the WT MeV group than any vaccine group (Figure 2*A*). Hits to F and M proteins were mostly detected in the WT MeV group. Finally, few hits to L protein were seen in all groups, particularly day 0 and days 14–15 postvaccination of the 2rMeVH group. The day 0 antibody hits suggest potential background binding for this peptide.

We then looked at antibody hits for individual peptides (Figure 2*C*). Each peptide tile spans 56 amino acids and is labeled by its start and end positions within the protein. Only peptides that generated >20 hits in any rhesus macaques and any timepoint are shown. For MeV P/V peptides, groups infected with WT MeV or vaccinated with at least 1 dose of MMR had the highest hits to the P449-504 peptide, with a weaker response in the 1LAMV group for this peptide. For MeV N peptides, N477-525 was mostly targeted by the WT MeV group and all groups receiving at least 1 LAMV-containing vaccine, with variable levels of antibody hits to other N peptides. For MeV F peptides, only WT MeV animals generated any appreciable hits. Overall, these results suggest that WT MeV infection induces a greater breadth of antibody binding than LAMV or any rMeVH vaccine combinations.

Last, MuV and RuV epitope analysis demonstrated antibody binding to MuV fusion protein peptide tiles F57-112 and F85-140 in the WT MeV group (Supplementary Figure 2). To assess whether this cross-reactivity could be related to shared primary sequence features, the corresponding regions of the MeV F protein (UniProt P69353) and MuV F protein (UniProt P33481) were aligned using BLASTP. The MuV F85-140 tile contained short highly conserved sequences aligned with MeV F, including SRRHKRFAGV in MeV F107-116 and SRRHKRFAGI in MuV F97-106, differing only by a conservative V/I substitution, as well as an identical 10-amino acid sequence, AALGVATAAQ, in MeV F121-130 and MuV F111-120. Extension of the latter sequence to 13 amino acids retained 12 of 13 identical residues, with only a conservative I/V substitution. Thus, cross-reactivity observed between WT MeV and MuV F57-140 peptide tiles could be attributed to sequence identity. Cross-reactivity to RuV peptides in the WT MeV group was already detectable prior to immunization (day 0), thus likely nonspecific background binding.

### WT MeV Infection and rMeVH Vaccination, but Not MMR Vaccination, Induce H-Specific Peptide Hits

Epitope sites on the H protein comprise the N-terminal cytoplasmic and transmembrane domains, the 96-amino acid residue stalk, and the head domain spanning amino acids 181–617 [26]. To assess the magnitude and breadth of the H-specific antibody responses following vaccination and challenge with WT MeV, we also quantified H protein (Figure 2*B*) and peptide tile (Figure 2*C*) targets.

While MMR vaccinations alone did not induce H-specific antibody binding in the VirScan platform, 2 doses of rMeVH vaccine expectedly resulted in high levels of H-specific binding postvaccination (Figure 2*B*), confirming prior ELISA findings [20]. Average H protein hits in the 2rMeVH group were higher between day 28 and days 84–89 postvaccination than in the WT MeV group. A single booster dose of rMeVH after a primary MMR vaccination induced a mild increase in H-specific antibody hits at days 84–89, although levels dropped to prevaccination baseline by days 112–176 postvaccination (Figure 2*B*).

Interestingly, the WT MeV, 2rMeVH, and 1LAMV groups targeted distinct H protein epitopes (Figure 2*C*): The 2rMeVH group had hits to the peptide H337-392 with lower levels to H365-420 and H393-448, while WT MeV animals had hits primarily to H1-56 (not contained in the rMeVH vaccine), with lower levels to H113-196. A single intratracheal dose of LAMV resulted in low responses to H589-620 at days 14–15 and H1-56 at day 28 postvaccination. Altogether, the 2rMeVH, WT MeV, and 1LAMV, but not the 1MMR/1rMeHM or 2MMR groups, elicited considerable H protein hits to diverse epitope regions.

### WT MeV Challenge Increases MeV-Specific VarScores for All Vaccinated Groups

Approximately 6 months after vaccination, 3 vaccine groups (2MMR, 2rMeVH, and 1MMR/1rMeVH) were intratracheally challenged with WT MeV. As previously reported, no vaccinated animal developed evidence of systemic measles symptoms and were considered protected from disease. However, both vaccinated and WT-infected animals generated high titers of MeV-specific neutralizing antibodies [20]. All WT MeV–challenged and MeV-vaccinated groups experienced an increase in MeV VarScore and PPH from prechallenge baseline, peaking between days 14–15 and day 28 postchallenge (peak VarScores of 2MMR at days 14–15, 2.94 ± 1.55; 2rMeVH at day 28, 1.68 ± 1.01; 1MMR/1rMeVH at days 14–15, 2.54± 0.92) (Figure 3*A* and Supplementary Figure 3). The increase in VarScore trended higher in the 2MMR group at days 14–15 and day 28 postchallenge compared to the 2 rMeVH group. Altogether, these data suggest that all groups responded to the MeV infection with a boost from baseline anti-MeV antibody titers.

Of interest, in macaques who had received the MMR vaccine, RuV VarScores were stable from day 0 to days 84–89 postchallenge, as supported by longitudinal ELISA data (Supplementary Figure 4), while MuV VarScores were stable or increased by days 84–89 postchallenge (Figure 3*B*), likely representing cross-reactivity between MeV and MuV antibodies (Figure 3*B*).

### Breadth of Epitope Binding Increases Following WT MeV Challenge in Vaccinated Macaques

Following WT MeV challenge, all 3 vaccinated groups had increased antibody hits to MeV proteins P/V, N, and H, compared to their postvaccination timepoints (Figure 4*A* and 4*B*). At the epitope/peptide level, both MMR-vaccinated groups (2MMR and 1MMR/1rMeVH) saw an increase in hits to MeV P/V peptides, with the greatest breadth observed in the 2MMR group (Figure 4*C*). All vaccine groups saw robust increases in hits to the P449-504 peptide tile, for which both MMR-vaccinated groups had preexisting antibodies.

In the 2rMeVH group, a modest increase in hits to H337-392 was observed, although these increases were lower than what was observed postvaccination. Likewise, a mild increase in hits to this H peptide were seen in the 1MMR/1rMeVH group postchallenge. An increase in hits to H peptide H1-56 was also seen in the 2MMR group, following the pattern seen with WT MeV infection. In general, the H-specific epitope response after challenge was broader in groups receiving at least 1 rMeVH vaccine.

### VirScan Output Correlates With Traditional Measures of Humoral Response

To assess the extent to which VirScan results correlate with conventional measures of humoral responses, we determined the MeV-specific IgG ELISA titers, avidity assay indices, and PRNT titers from WT MeV– (previously published [20]) and LAMV-infected rhesus macaques (Supplementary Figure 4). A correlational analysis of MeV VarScore with MeV-specific IgG ELISA titers (Figure 5*A*), avidity assay indices (Figure 5*B*), and PRNT titers (Figure 5*C*), using previously generated data from WT MeV–infected animals [20, 23], demonstrated that MeV VarScores were positively correlated with all 3 measures of MeV-specific humoral responses, with WT MeV–infected animals mounting stronger humoral responses than LAMV-infected animals over time. Likewise, RuV VarScores were also positively correlated with the corresponding ELISA titers (Supplementary Figure 5). These results suggest that VarScores can provide a useful correlate of protective immunity in MeV and RuV infections.

## DISCUSSION

Using VirScan, we demonstrate that distinct antibody repertoires arise following MeV vaccination versus WT MeV infection in a macaque model. Macaques receiving LAMV (intratracheal or subcutaneous) developed antibodies primarily targeting a single peptide in the P and N proteins, whereas WT MeV infection produced broader epitope reactivity across P/V, N, H, and F. Macaques vaccinated with prefusion-stabilized H protein generated H-specific antibodies with epitope profiles distinct from LAMV vaccination and maintained distinct profiles even after WT infection. Last, VirScan output correlated with traditional measures of humoral response in humans and macaques.

The greatest peptide hits in LAMV- and WT MeV–infected macaques were in the N and P proteins, consistent with literature in humans [13]. The measles transcriptional gradient plays a role in antigen quantity in both WT and LAMV infection, as genes closer to the 3′ end of the transcript (3′-N-P-M-F-H-L-5′) are transcribed up to 250 times more [27, 28]. The greater breadth of epitope reactivity seen after WT MeV infection compared to LAMV vaccination may be explained by the amount of antigen present during WT infection, as LAMV replication in peripheral blood mononuclear cells is restricted and systemic spread is limited [29]. Macaques receiving a single intratracheal dose of LAMV had high levels of N protein antibody reactivity compared to those receiving subcutaneous MMR, likely reflecting more efficient LAMV replication in respiratory epithelial cells [29, 30].

MeV-neutralizing antibodies are primarily directed to the H protein, yet there was little to no H protein reactivity in macaques receiving an LAMV-containing vaccine. Instead, high levels of H-specific antibody were identified in macaques that received 2 doses of rMeVH or were challenged with WT MeV. This may be related to higher levels of H antigen in the purified rMeVH vaccine. Further, patterns of H peptide reactivity differed between natural infection and rMeVH vaccination. Natural infection or LAMV vaccination followed by challenge resulted in antibody reactivity to peptide H1-56. The rMeVH vaccine did not contain this peptide, but even following WT MeV challenge the rMeVH-vaccinated macaques maintained reactivity to peptide H337-392. This may represent an example of immunological imprinting, whereby the immune system preferentially uses memory responses from the first exposure to the antigen even after subsequently encountering a variant of the antigen [31].

The rMeVH vaccine–induced antibodies to a peptide containing a portion the immunodominant “hemagglutinating and noose epitope,” which contains 3 cysteines and a loop formed by a disulfide bond between Cys-386 and Cys-394. This conformational epitope is the target of several protective and neutralizing monoclonal antibodies [32, 33], but since disulfide linkages and glycosylation are not formed within the T7 phage display system used here, it is very unlikely that this conformational epitope is preserved [22]. WT-infected and LAMV-vaccinated and challenged macaques developed antibodies mainly targeting the H1-56 peptide containing the cytoplasmic tail and transmembrane domain [26]. As Muñoz-Alía et al showed that the neutralizing antibody response to MeV targets numerous codominant epitopes [10], this might explain why both rMeVH- and LAMV-vaccinated macaques controlled infection despite generating distinct antibody profiles [20].

VirScan analysis could be useful for describing biomarkers for vaccine efficacy or correlates of protection, as we have demonstrated that the VarScore of WT-infected macaques was positively correlated with traditional measures of humoral reactivity. Thus, VarScore may be valuable in predicting protective responses following vaccination or infection, and in high-throughput screening of patient samples against existing or novel vaccine or virus strains.

Analysis of MuV and RuV VarScores and antibody epitope binding also yielded insights. In the analysis of RuV antibody responses upon WT MeV challenge, both RuV-specific ELISA titers and VarScores were stable longitudinally in MMR-vaccinated macaques. Since WT MeV infection is known to cause immune amnesia—a reduction in antibodies against previously encountered pathogens [21]—this result suggests that vaccine-induced MeV immunity might be sufficient to ameliorate long-term immune dysfunction. MuV VarScore increased in WT-infected macaques, which was attributed to the high antibody binding to MuV F protein at the peptide tile of F57-112 and F85-140. As MeV and MuV anti-F antibodies are not known to be cross-protective, these signals likely reflect conserved linear sequence motifs rather than functional immunity.

As discussed, this study is limited by the use of short, linear peptides, which may lack natural conformation. Nonetheless, given the excellent correlation to immune protective antibody responses, the approach provides a first-pass screen to identify epitopes of interest and differential peptide targeting patterns. In addition, study group sizes were limited and variability between individual animals may have affected the results. Future work should explore functional differences in epitope targeting between LAMV and rMeVH, including potential evidence of immune imprinting.

In summary, we integrated VirScan data with clinical outcome measures to characterize the breadth and specificity of humoral immunity elicited by different vaccine strategies before and after WT MeV challenge. The data support the hypothesis that vaccine platforms shape epitope targeting, with implications for immunological memory and vaccine efficacy.

## Supplementary Data

Supplementary materials are available at *The Journal of Infectious Diseases* online (http://jid.oxfordjournals.org/). Supplementary materials consist of data provided by the author that are published to benefit the reader. The posted materials are not copyedited. The contents of all supplementary data are the sole responsibility of the authors. Questions or messages regarding errors should be addressed to the author.
