# Supplementary material for: Distinct Humoral Response Development to Measles Virus Across Vaccine Platforms
Source: J Infect Dis. 2026 Jun 17;234(4):e765–76. doi: 10.1093/infdis/jiag309 (PMC13668335; doi:10.1093/infdis/jiag309)
Supplement: Supplementary file 1 [file jiag309_supplementary_data.zip › Supplemental_figures_VirScan_5Jan2026.docx]

**Supplemental Figure 1** – Longitudinal antibody reactivities to measles (MeV), mumps (MuV) and rubella virus (RuV) in macaques after vaccination, as measured by percentage peptide hits (PPH). (**A**) PPH to MeV, MuV and RuV in macaques vaccinated with one dose of LAMV at d0 (1LAMV, n=3), 2 doses of MMR or rMeVH at both d0 and d42 (2MMR or 2rMeVH respectively, n=4 per group) or 1 dose of MMR at d0 and 1 dose of rMeVH at d42 (1MMR/1rMeVH, n=4). 5 timepoints (d0, d14/15, d28, d84/89 and d112/168/176) were captured per group. The horizontal dotted line represented the average longitudinal VarScores from each of the three viruses in the sham control group to compute a threshold value, above which a positive infection was identified. The gray lines represent values from each macaque, and the red line indicates the average values from all macaques in each group. (**B**) Statistical significance between macaque groups per timepoint was evaluated by Wilcoxon rank-sum test, with nonsignificant results shown by missing asterisks. **P* < 0.05; ***P* < 0.01; DPV, days post-vaccination.

**Supplemental Figure 2** – Longitudinal average peptide hits to MuV and RuV proteins and peptide tiles after vaccination with different modalities. Longitudinal average peptide hits to (**A**) all MuV and RuV proteins and (**B**) selected viral peptides in different vaccinated groups, where viral peptide tiles were selected if average hit-fold change was higher than 10 in any timepoint in any macaque. 5 timepoints (d0, d14/15, d28, d84/89 and d112/168/176) were captured per group. hfc, hit-fold change. DPV, days post vaccination.

**Supplemental Figure 3** – Longitudinal antibody reactivities to MeV in previously-vaccinated macaques after WTMeV challenge, as measured by PPH. PPH to MeV in previously-vaccinated macaque groups: 2MMR, 2rMeVH and 1MMR/1rMeVH. Longitudinal data from WTMeV-infected macaques was also included for comparison. The horizontal dotted line represents the average longitudinal VarScores for MeV in the sham control group in Figure 1 to compute a threshold value, above which a positive infection was identified. DPC, days post-challenge; PPH, percentage peptide hits.

**Supplemental Figure 4** – Longitudinal MeV-specific IgG ELISA titers, avidity index and PRNT between WT MeV and LAMV groups. Longitudinal MeV-specific IgG **(A)** ELISA titers, **(B)** avidity index and **(C)** PRNT between WT MeV and LAMV groups. Statistical significance between groups per timepoint was evaluated by Wilcoxon rank-sum test, with nonsignificant results shown by missing asterisks or NS. **P* < 0.05; ***P* < 0.01. RM, rhesus macaques.

**Supplemental Figure 5** – Longitudinal rubella-specific IgG ELISA titers and its correlation to VarScore. **(A)** Longitudinal rubella-specific IgG ELISA titers post vaccination and WT MeV challenge in two macaque groups receiving at least one dose of MMR (1MMR/1rMeVH and 2MMR). DPV-D228/233 indicated DPC D0. Data from one macaque was removed from the analysis of 1MMR/1rMeVH and 2MMR groups due to elicitation of markedly lower and higher titers, respectively. **(B)** Spearman correlation was computed between RuV VarScore and ELISA titers from the 6 macaques in A from 4 longitudinal timepoints (d14/15, d28, d84/89 and d112/168/176). BL, background level at D0; DPV, days post vaccination; DPC, days post challenge.
